# Food Addiction Is Associated with Irrational Beliefs via Trait Anxiety and Emotional Eating

**DOI:** 10.3390/nu11081711

**Published:** 2019-07-25

**Authors:** Laurence J. Nolan, Steve M. Jenkins

**Affiliations:** Department of Psychology, Wagner College, 1 Campus Rd., Staten Island, NY 10301, USA

**Keywords:** food addiction, irrational beliefs, emotional eating, anxiety, food misuse

## Abstract

Irrational beliefs (IB) are believed, in cognitive behavioral therapies, to be a prime cause of psychopathologies including anxiety, depression, problem eating, and alcohol misuse. “Food addiction” (FA), which has been modeled on diagnostic criteria for substance use disorder, and emotional eating (EE) have both been implicated in the rise in overweight and obesity. Both FA and EE are associated with anxiety. Thus, in the present study, the hypothesis that IB is associated with FA and with EE was tested. Furthermore, possible mediation of these relationships by trait anxiety and depression (and EE for IB and FA) was examined. The responses of 239 adult participants to questionnaires measuring FA, IB, EE, depression, trait anxiety, and anthropometrics were recorded. The results revealed that IB was significantly positively correlated with FA and EE (and depression and trait anxiety). Furthermore, only EE mediated the effect of IB on FA and this was not moderated by BMI. Finally, trait anxiety (but not depression) mediated the effect of IB on EE. Exploratory analysis revealed a significant serial mediation such that IB predicted higher FA via elevated trait anxiety and emotional eating in that order. The results of this study suggest that IB may be a source of the anxiety that is associated with EE and FA and suggest that clinicians may find IB a target for treatment of those persons who report experiences of EE and FA. IB may play a role in food misuse that leads to elevated BMI.

## 1. Introduction

“Food addiction” (FA) has been suggested as a factor in the increased prevalence of overweight and obesity. Proponents of FA suggest that some energy-dense highly palatable foods (or specific additives to foods such as salt or refined sugar) generate addiction-like behaviors in those who consume them [1]. FA, as measured by the Yale Food Addiction Scale (YFAS), is associated with binge eating behavior (BED) [2,3,4], bulimia nervosa [5], night eating syndrome [6], and with elevated BMI even in the absence of BED [4,7,8]. The FA concept is not without controversy. Some critics prefer an alternate description that focuses on the behavior (i.e., “eating addiction”) and suggest there is little evidence for an addicting substance in food. They instead suggest that overeating may be a form of habitual food “abuse” [9] or represent a possible food use disorder [10]. Others have suggested that there is not enough evidence yet to conclude that FA is a distinct entity that explains overeating [11]. Nonetheless, there has been significant interest in FA in the scientific community in recent years [12]. The YFAS, which was based on DSM IV substance dependence criteria, has now been updated to reflect the substance use disorder criteria described in the DSM-5 and dubbed the YFAS2 (which has since been provided in a shortened form [13]).

In cognitive behavioral therapies (CBT), irrational beliefs are believed to be a prime cause of psychopathologies including problem eating and addictive behavior. Ellis [14] and Beck [15] proposed that individuals often have habitual affect-eliciting thought patterns (referred to as irrational beliefs by Ellis) that can lead to dysfunctional emotional and/or behavioral responses. These irrational beliefs originate from a core process of perfectionism [14] or absolutist thinking [16] and the idea that one should be extremely upset when things go wrong and that it is crucial to be successful and approved of by everyone [15]. This absolutist thinking inevitably leads to anxiety and, in turn, may lead to irrational coping strategies such as substance use and uncontrolled eating typified by emotional eating and FA.

In a meta-analysis of 100 independent samples, irrational beliefs were found to be moderately correlated with psychological distress [17]. More specifically, irrational beliefs were associated with trait anxiety [18,19]. While Rohsenow and Smith [18] did not find a connection between irrational beliefs and depression (as measured by Minnesota Multiphasic Personality Inventory), in daily reports of mood over several months, there was an association of irrational beliefs and reports of depression. Others reported that irrational beliefs were related to depression as measured by the Beck Depression Inventory in a sample of women [20]. Mayhew and Edeleman [21] found that irrational beliefs were predictive of poor coping strategies and low self-esteem. Irrational beliefs have been associated with addictive behaviors such as drug misuse [22,23,24,25] and problem gambling (see [26] for review) although, in the gambling studies, irrational beliefs are often assessed using different measures than they are in studies of depression and anxiety.

Several studies (mostly involving samples of undergraduate women without eating disorder diagnoses) have reported a link between irrational beliefs and problem eating. Ruderman [27] reported that irrational beliefs were associated with dietary restraint (the cognitive control of food consumption as measured by the Revised Restraint Scale or RRS), particularly the concern with dieting subscale. Studies examining the relationship between irrational beliefs and subclinical eating disorder symptoms are more common. Irrational beliefs predicted a number of bulimia symptoms in undergraduate women [20,28,29]. In addition, irrational beliefs were found to be predictive of drive for thinness, body dissatisfaction, ineffectiveness, and poor interceptive awareness as measured by the Eating Disorders Inventory [21]. More recently, Tomotaki et al. [30] reported that obsession with eating, dieting, and obese-phobia were predicted by irrational beliefs. Irrational beliefs were found to be higher in women with high body dissatisfaction when compared to a group diagnosed with eating disorders and a group with low body dissatisfaction [31].

While irrational beliefs have been associated with dietary restraint, it has not been examined in relation to emotional eating. Emotional eating is generally viewed as a response to negative emotion or distress [32,33] or ego-threat [34], and has been associated with overeating, binge eating, bulimia nervosa, and obesity (see [32]). There is a positive association between emotional eating and anxiety in persons with obesity (but not in persons with a BMI between 18 and 25) [35] and in a sample of children and adolescents [36]. Irrational beliefs and depression were positively correlated in a sample of women [37]. Thus, irrational beliefs may be associated with emotional eating, possibly as the source of anxiety and/or depression.

The research findings described above suggest that irrational beliefs could predict FA and emotional eating. If they do, it is likely that there would be mediating variables. FA is positively correlated to depression in persons with obesity. Furthermore, FA has been associated with depression in persons with obesity [2,3] and in students and the general population [6,38]. FA has also previously been associated with emotional eating [1,2] and with anxiety [39]. The present study was conducted to determine whether the presence of irrational beliefs predicts higher FA symptoms. Furthermore, if such a relationship exists, it may be mediated by depression, trait anxiety, and/or emotional eating, and may depend on BMI. Absolutist irrational beliefs are predicted to produce psychological distress via activation of anxiety. Maladaptive responses such as emotional or uncontrolled eating (e.g., FA) may occur in response to this anxiety. Thus, the following hypotheses (H) were tested. Irrational beliefs and FA are positively correlated (H1). Furthermore, the effect of irrational beliefs on FA is mediated by trait anxiety, depression, and/or emotional eating (H1a). It was also hypothesized that there would be a positive relationship between irrational beliefs and emotional eating (H2) and that the effect of irrational beliefs on emotional eating is mediated by trait anxiety and/or depression (H2a). Finally, it was hypothesized that there would be a moderation of these relationships by BMI; that the effect of irrational beliefs would depend on the value of BMI (H3). Moderation by gender was also hypothesized but not examined in the present study due to the relatively low number of men in the sample.

## 2. Materials and Methods

### 2.1. Participants

The participants were 239 adults; the sample included individuals who identified as women (*n* = 176), men (*n* = 60), or as having non-binary gender (*n* = 3). The sample was mostly composed of undergraduate students. The mean age of participants was 20.72 years (SEM = 0.42; range = 18–57) and their mean BMI was 24.07 kg/m^2^ (SEM = 0.32; 5.4% had a BMI less than 18.5; 62.3% between 18.5 and 24.9; 20.9% were between 25 and 29.9; 11.3% ≥ 30). When asked in an open-ended question to describe their ethnic or racial background, participants described themselves as having Arab (1.3%), African (6.3%), Asian (4.2%), European (73.2%), South Asian (3.8%), or more than one primary (4.6%) ancestry. In addition, some of the participants (6.7%) also indicated Hispanic ancestry. In total, 17.8% of the participants met the criteria for FA. In total, 20.9% of the sample had a depression score of 50 or higher suggesting the presence of depression. See Table 1 for mean scores on questionnaires.

### 2.2. Measures

#### 2.2.1. Food Addiction

FA was assessed by the Modified Yale Food Addiction Scale 2.0 (mYFAS2) which is designed to evaluate indications of “addiction” toward foods according to the DSM-5 criteria for substance use disorder but with fewer questions than the YFAS 2.0 [13]. The mYFAS2 has been validated against questionnaires that measure related constructs, and has a Kuder–Richardson’s alpha of 0.86. In the present study, Kuder–Richardson’s alpha was 0.77. The mYFAS2 has 13 items (11 for FA symptoms and 2 for distress) and is scored by counting the number of diagnostic criteria that are met. A person is considered to have FA when 3 or more of the criteria are met, and there is impairment or distress present. In analyses presented below, the mYFAS2 was entered as a continuous variable (number of symptoms).

#### 2.2.2. Irrational Beliefs

Irrational beliefs were measured by the Shortened General Attitude and Belief Scale (SGABS) [40]. The SGABS is a 26-item scale that uses Likert-type ratings with responses ranging from 1 (strongly disagree) to 5 (strongly agree). The SGABS has one rational beliefs subscale and six irrationality subscales (need for achievement, need for approval, need for comfort, demand for fairness, self-downing, and other downing) that are summed to create a total irrationality score (higher scores indicate stronger irrational beliefs). Several instruments are available to measure irrational beliefs, some of which may be more sensitive to affect than to cognitions (for a review see [41]). The SGABS is based on the Ellis model of psychopathology [41] and its score has been shown to be less affected by mood than some previous measures [40]. The reliability for this sample was very good (Cronbach’s α = 0.86).

#### 2.2.3. Eating Styles

Eating styles were measured by the Dutch Eating Behavior Questionnaire (DEBQ) which contains three subscales: restrained eating (DEBQr), emotional eating (DEBQe), and external eating (DEBQx) [42]. All 33 questions are rated on a 5-point Likert-type scale with ‘‘never’’ and ‘‘very often’’ as the anchors. The restraint (cognitive restraint of eating) and external eating (eating in the presence of external cues) scales each have 10 items while the DEBQe contains 13 items. Score for each subscale is the mean rating. In the present sample, the Cronbach’s α for the DEBQe, DEBQr, and DEBQx were 0.94, 0.79, and 0.94 respectively.

#### 2.2.4. Trait Anxiety

Trait anxiety was measured using the State-Trait Anxiety Inventory for Adults (STAI) [43]. The STAI differentiates between trait anxiety and state anxiety with 40 items (4-point Likert-type scale) regarding how participants generally feel (trait) and how they feel at this moment (state). Only the trait measure was used in the statistical analysis. In the present study, the Cronbach’s α was 0.93.

#### 2.2.5. Depression

Depression was assessed using the Self-report Depression Scale (SDS) [44]. The SDS score is the sum of responses to 20 questions to which the participant responds on a 4-point Likert-type scale. The total score can range from 20 to 80 with most depressed persons scoring between 50 and 69 [45]. The Cronbach’s α for the SDS was 0.87 for this sample.

### 2.3. Procedure

The hypotheses were pre-registered with the Open Science Framework after data collection had commenced but prior to examination of the data (doi: 10.17605/OSF.IO/QWSRD). The procedure was approved by the Wagner College Human Experimentation Review Board (code F18–10).

All participants completed questionnaires using an online platform (Qualtrics, Provo, UT, USA). Questionnaires were presented in randomized order after informed consent was obtained. Questions regarding age, height and weight, gender, and ethnicity were presented after questionnaires. All participants were debriefed as to the purpose of the study after the survey was completed. In the debriefing, none of the participants appeared to be aware of the study’s purpose. Students participated in groups at scheduled times in a computer laboratory as one way to complete a research requirement for an introductory psychology course (*n* = 174). Other participants were recruited via the university daily email bulletin and via a link (which took them to the same website on the university server as that used by the students) posted on Facebook. These participants were entered into a lottery to win one of four $25 gift cards if they wished.

### 2.4. Data Analysis

To ensure quality of data, records were screened for inappropriate responses to open-ended questions, lack of response variation (e.g., giving the same answer to all questions), and unusually short survey completion times.

Statistical analysis was performed using IBM SPSS (version 24). The variables were first correlated to establish whether there was a relationship between irrational beliefs and FA and whether each was correlated to trait anxiety, depression, eating styles, and BMI. Then, mediation multiple regression analysis was conducted using the PROCESS plug-in for SPSS (release 2.16.3) [46] using 5000 bootstrap samples. Variables were mean-centered and heteroscedasticity-consistent standard errors were used. Residuals were checked for stochasticity prior to the analysis. The unstandardized beta coefficients (B), confidence intervals (CI) and adjusted R^2^ are reported. Planned analysis included testing whether the relationship between irrational beliefs and FA was mediated by emotional eating, trait anxiety, and/or depression and whether these would be moderated by BMI. That is, the causal hypothesis was that irrational beliefs produce an elevation in FA by increasing trait anxiety, depression, and/or emotional eating. Furthermore, the mediation of the effect of irrational beliefs on emotional eating by trait anxiety and/or depression was also examined (the hypothesis that irrational beliefs cause elevated emotional eating by increasing trait anxiety and/or depression). The results of these analyses led to an exploratory analysis of the indirect pathway between irrational beliefs, trait anxiety, depression, emotional eating, and FA in that order (serial mediation). This final analysis tested the hypothesis that irrational beliefs increase FA symptoms via a pathway from higher trait anxiety and/or depression to higher emotional eating.

## 3. Results

### 3.1. Confirmatory Analyses

#### 3.1.1. Correlations

To examine the relationships among the questionnaire variables and test hypotheses H1 and H2, Pearson correlation coefficients were performed. The results indicated that irrational beliefs were positively correlated with all measures (most strongly with depression and trait anxiety; see Table 1). Correlation coefficients indicated that body mass index was significantly positively correlated only with dietary restraint and FA and not with irrational beliefs or other measures (see Table 2).

#### 3.1.2. Multiple Regression Mediation Analysis

##### Multiple Mediation of the Effect of Irrational Beliefs on FA

**Figure 1:**
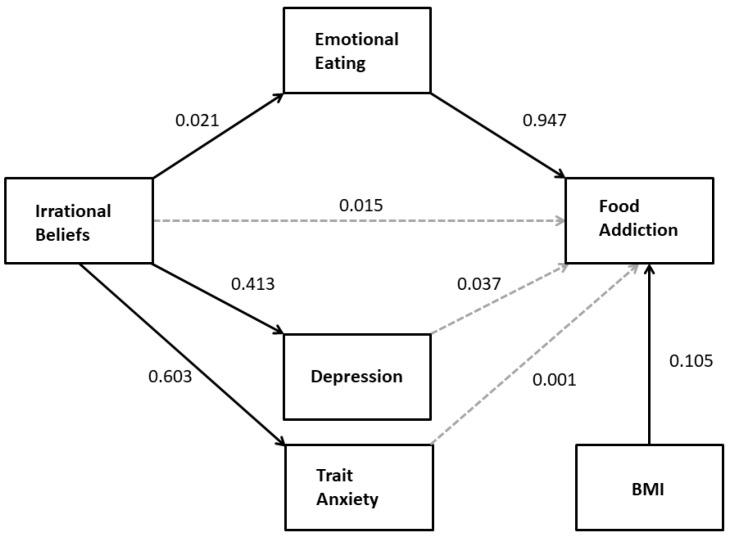
The relationship between irrational beliefs and food addiction (FA) is mediated by emotional eating but not depression or trait anxiety. Solid arrows indicate statistically significant regression coefficients. Nonsignificant BMI effects on mediators are not included for simplicity.

Multiple mediation analysis (PROCESS model 4) was used to determine whether the effect of irrational beliefs on FA was mediated by depression, trait anxiety, and/or emotional eating (H1a). BMI was included as a covariate because of its correlation with the criterion variable, FA. The results (see Figure 1) indicated that, although irrational beliefs significantly predicted elevated scores on depression, trait anxiety, and emotional eating measures, only emotional eating mediated the effect; there was a significant indirect effect of irrational beliefs on FA through emotional eating (*B* = 0.02; 95%CI: 0.010–0.034) but not though trait anxiety (*B* = 0.00; 95%CI: −0.021–0.021) or depression (*B* = 0.02; 95%CI: −0.001–0.035). In the mediation model, there was no significant direct effect of irrational beliefs on FA (*B* = 0.02, *t* = 1.17, *p* = 0.242; 95%CI: −0.010–0.040). The total effect was statistically significant (*B* = 0.05, *t* = 4.20, *p* < 0.001; 95%CI: 0.027–0.074).

##### Moderated Mediation by BMI of the Effect of Irrational Beliefs on FA

**Figure 2:**
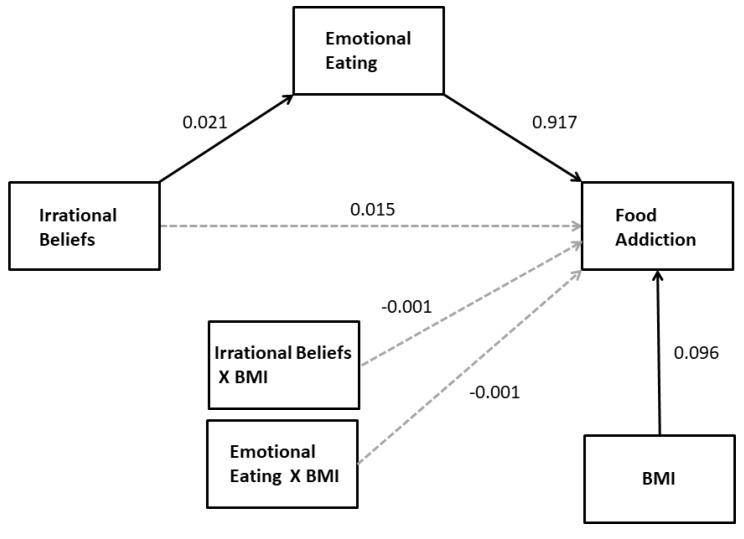
The statistical model for the moderated mediation of the effect of irrational beliefs on FA with BMI as moderator. Solid arrows indicate statistically significant regression coefficients. Nonsignificant mediator variables and BMI effects on mediators are not shown for simplicity.

In order to examine whether BMI moderated the direct and indirect (e.g., through emotional eating) effects of irrational beliefs on FA (H3), BMI was added to the model presented above as a moderator (a moderated mediation model, PROCESS model 15). The results of this analysis again showed emotional eating to be the only significant mediator of the effect of irrational beliefs on FA and indicated that there were no significant interactions between BMI and other effects on FA (see Figure 2). There was no significant conditional direct effect of irrational beliefs on FA at mean-1SD, mean, and mean+1SD values of BMI. In addition, there was a statistically significant conditional indirect effect of irrational beliefs on FA at mean-1SD BMI (95%CI: 0.008–0.037), mean BMI (95%CI: 0.009–0.034) and mean+1SD BMI (95%CI: 0.007–0.038); each coefficient was virtually identical (*B* = 0.019). The index for moderated mediation was not statistically significant (95%CI: −0.0017–0.0017). Finally, there was no moderation of the nonsignificant mediated effects of depression and trait anxiety on FA.

##### Multiple Mediation of the Effect of Irrational Beliefs on Emotional Eating

**Figure 3:**
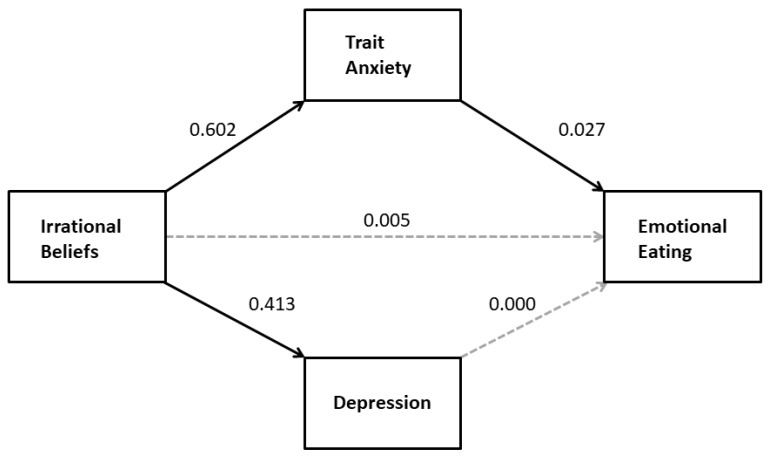
The relationship between irrational beliefs and emotional eating is mediated by trait anxiety but not depression. Solid arrows indicate statistically significant regression coefficients.

Mediation analysis (PROCESS model 4) was used to determine whether the effect of irrational beliefs on emotional eating was mediated by depression and/or trait anxiety (H2a). The results indicated that, although irrational beliefs significantly predicted elevated scores on depression and trait anxiety, only the latter mediated the effect of irrational beliefs on FA (see Table 3). There was a significant indirect effect of irrational beliefs on emotional eating through trait anxiety (*B* = 0.02; 95%CI: 0.006–0.027) but not through depression (*B* = 0.00; 95%CI: −0.007–0.007; see Figure 3). In the mediation model, there was no significant direct effect of irrational beliefs on emotional eating (*B* = 0.01, *t* = 0.83, *p* = 0.406; 95%CI: −0.007–0.016). The total effect was statistically significant (*B* = 0.02, *t* = 4.69, *p* < 0.001, 95%CI: 0.012–0.030).

### 3.2. Exploratory Analyses: Serial Mediation of the Effect of Irrational Beliefs on FA

**Figure 4:**
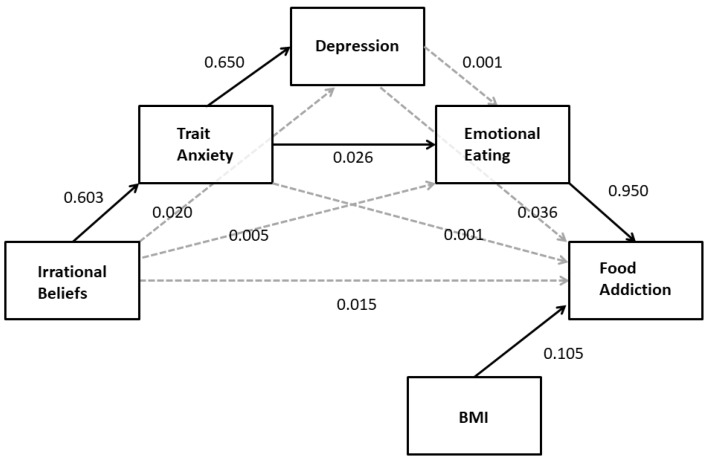
The relationship between irrational beliefs and FA is mediated by the serial pathway through trait anxiety and emotional eating. Solid arrows indicate statistically significant regression coefficients. Nonsignificant BMI effects on mediators are not included for simplicity.

The results presented above indicate a strong relationship between irrational beliefs and depression and trait anxiety but neither predicted FA. Because irrational beliefs are associated with emotional eating via trait anxiety and anxiety and depressed mood have been associated with emotional eating, an additional serial mediation analysis was conducted. Based on the results of the planned correlational and mediation analyses presented above, the hypothesis that the indirect pathway between irrational beliefs and FA would include trait anxiety and depression was examined. Specifically, it was proposed that irrational beliefs would increase trait anxiety which, in turn, would increase depression. Depression and/or trait anxiety was expected to increase emotional eating which, in turn, would increase higher food addiction. BMI was included as a covariate in the model because of its correlation with FA. The results of this analysis (PROCESS model 6; see Table 4) largely supported this hypothesis with the exception that depression was not associated with elevated emotional eating. As depicted in Figure 4, there was a significant indirect effect of irrational beliefs on food addiction through trait anxiety and emotional eating (*B* = 0.02; 95%CI: 0.007–0.028). While trait anxiety was a significant predictor of higher depression score (*B* = 0.65, *t* = 15.56, *p* < 0.001; 95%CI: 0.568–0.732), the indirect path including depression was not statistically significant (*B* = 0.00; 95%CI: −0.006–0.007).

## 4. Discussion

The results show that, as hypothesized, FA and emotional eating were each positively associated with irrational beliefs. The results of this study are consistent with cognitive behavioral theory and confirm previous findings using different measures of both irrational beliefs and psychopathology that irrational beliefs are associated with elevated trait anxiety and depression. While irrational beliefs did predict higher trait anxiety, depression, and emotional eating, only emotional eating mediated the effect of irrational beliefs on FA. This mediated effect was the same across values of BMI; thus, contrary to prediction, we were unable to show that BMI moderated the mediation of the effect of irrational beliefs on FA by emotional eating. The results also confirmed that the effect of irrational beliefs on emotional eating was mediated by trait anxiety. These findings suggested examination of a serial mediation which found that the indirect effect of irrational beliefs on FA also included trait anxiety. That is, the only significant pathway indicated that irrational beliefs increased trait anxiety which, in turn, increased emotional eating, which finally led to higher number of FA symptoms. The findings that trait anxiety and not depression mediated the associations between irrational beliefs and FA and emotional eating are consistent with the suggestion that anxiety is more important than depression of symptoms of problem eating [see 29]. Finally, we have confirmed that irrational beliefs were positively associated with restrained eating (using a measure other than the RRS) and have found that irrational beliefs were positively correlated with external eating (eating in the presence of food) both of which have been implicated in problem eating and control of body weight. The role of irrational beliefs in restraint and external eating warrants additional exploration as these relationships may be mediated by anxiety or depression.

The research method employed does not allow for causal relationships to be determined with certainty. However, mediation analysis depends upon a theory of causality in order to determine the order of placement of variables in statistical models. In the present analysis, the assumptions were that irrational beliefs are the cause of the psychopathologies and coping behaviors measured because, in CBT, irrational beliefs are considered prime sources of psychopathology. In traditional psychotherapy, it was often believed that activating events (i.e., negative occurrences) result in emotional consequences (i.e., psychological distress). However, according to Ellis [47], this theory is inaccurate or at best incomplete. In the research that led to the development of Rational Emotive Behavior Therapy (REBT), Ellis found that the emotional consequences of an activating event were primarily dictated by the belief a person holds about the activating event. For example, if an employee is reprimanded by an employer for a minor mistake, she or he could think "I am incompetent and will surely be fired from my job, and then no one will ever want to hire me!" The emotional consequence of such a belief would most likely be a significant level of anxiety. If that same person had the more rational thought "It is unfortunate that I made a mistake, but I am human so it will happen sometimes. It is highly unlikely that I will be fired if I make a minor mistake once in a while.", the emotional consequence would likely be one of mild concern or annoyance. Hence, one’s emotional state is usually the result of how he or she interprets an event, rather than the event itself. Ellis [47] found that individuals who frequently interpret reality from a distorted, or irrational, perspective are likely to have anxiety or depressive disorders. He also found that when people are emotionally disturbed, they seek out ways to cope with the distress. Coping mechanisms can be adaptive (i.e., make appropriate changes, practice acceptance, exercise, etc.) or maladaptive (i.e., substance use, self-harm, uncontrolled eating). Indeed, some persons may eat to regulate mood and escape anxiety [48,49].

Irrational beliefs-based uncontrolled eating may not necessarily lead to weight gain; in the present study, irrational beliefs were not correlated with BMI. The research on the relationship between irrational beliefs and alcohol consumption indicates that irrational beliefs are associated with problems with alcohol use and not amount of alcohol used [22,23] or frequency of use or getting drunk [24]. Furthermore, perceived lack of control over alcohol use is correlated with irrational beliefs [24]. This is interesting in relation to FA as, unlike alcohol consumption, everyone needs to eat food but those with FA constitute a subset of people who have problem eating who often feel that they cannot control eating. Indeed, lack of control over eating is the most commonly reported FA symptom [50]. Irrational beliefs may lead to FA; FA is common in those with high BMI [7]. It is important to determine whether irrational beliefs are associated with energy consumption from food which may lead to higher BMI in some people. Recent findings suggest that psychological distress is associated with elevated BMI via higher FA and emotional eating [51]. Irrational beliefs may be a source of that psychological distress.

This study has several limitations. While mediation models often use causal wording (i.e., direct and indirect “effects”), the results are correlational and the direction of effect speculative. The sample is composed mostly of students who report having normal body weight. While students are of interest in the study of anxiety and other psychopathology due to high rates of anxiety, depression [52,53] and problem eating [54], their overrepresentation in the present study may limit the generalizability of the findings. Furthermore, while significant, the conditional effects are somewhat weak which may be due to the relatively low percentage of people with BMI greater than 25. In addition, while nearly a fifth of the sample meets the criterion for FA, the number of symptoms is rather low in the sample as a whole. Given the association of emotional eating and FA in persons with high BMI, the relationships reported here would be expected to be higher in those with high BMI. 

## 5. Conclusions

In conclusion, these results suggest for the first time that irrational beliefs may underlie problem eating such as emotional eating and FA via trait anxiety. Additional research may consider whether anxiety sensitivity, the fear that somatic arousal leads to catastrophic consequences, more strongly mediates the relationship between irrational beliefs and FA than trait anxiety. Anxiety sensitivity is distinct from trait anxiety and has been related to substance misuse and emotional eating, particularly in those with high BMI [55]. The results of the present study also suggest that irrational beliefs may be an appropriate target for clinicians when treating problem eating; CBT is an effective approach in treatment of addictive behaviors and problem eating [56].

## Figures and Tables

**Table 1:** Pearson correlation coefficients among and descriptive statistics for the questionnaire measures.

Measure	SGABS	DEBQe	DEBQx	DEBQr	SDS	mYFAS2	STAI
SGABS		0.298 ^4^	0.166 ^1^	0.242 ^2^	0.498 ^4^	0.282 ^3^	0.601 ^4^
DEBQe			0.530 ^4^	0.388 ^4^	0.338 ^4^	0.485 ^4^	0.422 ^4^
DEBQx				0.219 ^2^	0.039	0.409 ^4^	0.057
DEBQr					0.182 ^1^	0.328 ^4^	0.240 ^2^
SDS						0.342 ^4^	0.800 ^4^
mYFAS2							0.362 ^4^
Mean	59.09	2.38	3.06	2.81	40.91	1.62	43.87
SEM	0.79	0.06	0.04	1.01	0.66	0.14	0.79

^1^*p* ≤ 0.01, ^2^ *p* ≤ 0.001, ^3^ *p* < 0.0001, ^4^ *p* < 0.00001. SGABS: Shortened General Attitude Belief Scale. DEBQe: Dutch Eating Behavior Questionnaire Emotional Eating. DEBQx: Dutch Eating Behavior Questionnaire External Eating. DEBQr: Dutch Eating Behavior Questionnaire Restrained Eating. SDS: Self-report Depression Scale. mYFAS2: Modified Food Addiction Scale 2.0. STAI: State-Trait Anxiety Inventory.

**Table 2:** Pearson correlation coefficients among the questionnaire measures and body mass index (BMI).

Measure	SGABS	DEBQe	DEBQx	DEBQr	SDS	mYFAS2	STAI
BMI	−0.020	0.082	0.037	0.161 ^1^	−0.024	0.269 ^2^	0.032

^1^*p* < 0.05, ^2^ *p* < 0.0001. SGABS: Shortened General Attitude Belief Scale. DEBQe: Dutch Eating Behavior Questionnaire Emotional Eating. DEBQx: Dutch Eating Behavior Questionnaire External Eating. DEBQr: Dutch Eating Behavior Questionnaire Restrained Eating. SDS: Self-report Depression Scale. mYFAS2: Modified Food Addiction Scale 2.0. STAI: State-Trait Anxiety Inventory.

**Table 3:** Multiple mediation model predicting DEBQe from SGABS. Total effect: R^2^ = 0.181, F(3,235) = 15.98, *p* < 0.0001.

**Predictor**	**Mediator: STAI** **R^2^ = 0.362, F(1,237) = 148.52, *p* < 0.0001**			**95%** **Confidence Interval**	
	**Coeff.**	**SE**	***p***	**Lower**	**Upper**
SGABS	0.602	0.049	<0.0001	0.505	0.670
	**Mediator: SDS** **R^2^ = 0.248, F(1,237) = 75.85, *p* < 0.0001**				
SGABS	0.413	0.048	<0.0001	0.320	0.507
	**Criterion: DEBQe** **R^2^ = 0.182, F(3,235) = 15.98, *p* < 0.0001**				
SGABS	0.005	0.006	0.406	−0.007	0.016
STAI	0.027	0.009	0.002	0.010	0.044
SDS	0.000	0.008	0.989	−0.017	0.016

STAI: State-Trait Anxiety Inventory. SDS: Self-report Depression Scale. DEBQe: Dutch Eating Behavior Questionnaire Emotional Eating. SGABS: Shortened General Attitude Belief Scale.

**Table 4:** Serial mediation analysis predicting mYFAS2 from SGABS. Total effect: R^2^ = 0.155, F(2,236) = 16.93, *p* < 0.0001.

**Predictor**	**Mediator: STAI** **R^2^ = 0.363, F(2,236) = 72.58, *p* < 0.0001**			**95%** **Confidence Interval**	
	**Coeff.**	**SE**	***p***	**Lower**	**Upper**
SGABS	0.603	0.050	<0.0001	0.504	0.702
BMI	0.109	0.171	0.525	−0.227	0.445
	**Mediator: SDS** **R^2^ = 0.643, F(3,235) = 180.88, *p* < 0.0001**				
SGABS	0.020	0.044	0.656	−0.067	0.106
STAI	0.653	0.042	<0.0001	0.570	0.735
BMI	−0.100	0.083	0.229	−0.263	0.063
	**Mediator: DEBQe** **R^2^ = 0.186, F(4,234) = 13.03, *p* < 0.0001**				
SGABS	0.005	0.006	0.387	−0.007	0.017
STAI	0.026	0.009	0.004	0.008	0.043
SDS	0.001	0.008	0.933	−0.016	0.017
BMI	0.013	0.120	0.299	−0.011	0.036
	**Criterion: mYFAS2** **R^2^ = 0.334, F(5,233) = 14.51, *p* < 0.0001**				
SGABS	0.015	0.013	0.242	−0.010	0.040
STAI	0.001	0.018	0.964	−0.035	0.037
SDS	0.037	0.022	0.092	−0.006	0.079
DEBQe	0.947	0.194	<0.0001	0.565	1.328
BMI	0.105	0.026	.0001	0.054	0.157

STAI: State-Trait Anxiety Inventory. SGABS: Shortened General Attitude Belief Scale. SDS: Self-report Depression Scale. DEBQe: Dutch Eating Behavior Questionnaire Emotional Eating. mYFAS2: Modified Food Addiction Scale 2.0.
